# Whole-Body Regeneration in the Lobate Ctenophore *Mnemiopsis leidyi*

**DOI:** 10.3390/genes12060867

**Published:** 2021-06-05

**Authors:** Allison Edgar, Dorothy G. Mitchell, Mark Q. Martindale

**Affiliations:** The Whitney Laboratory for Marine Bioscience, 9505 N, Ocean Shore Blvd, St. Augustine, FL 32080-8610, USA; allison.edgar@whitney.ufl.edu (A.E.); dorothy.mitchell@whitney.ufl.edu (D.G.M.)

**Keywords:** ctenophore, regeneration, *Mnemiopsis leidyi*

## Abstract

Ctenophores (a.k.a. comb jellies) are one of the earliest branching extant metazoan phyla. Adult regenerative ability varies greatly within the group, with platyctenes undergoing both sexual and asexual reproduction by fission while others in the genus *Beroe* having completely lost the ability to replace missing body parts. We focus on the unique regenerative aspects of the lobate ctenophore, *Mnemiopsis leidyi*, which has become a popular model for its rapid wound healing and tissue replacement, optical clarity, and sequenced genome. *M. leidyi*’s highly mosaic, stereotyped development has been leveraged to reveal the polar coordinate system that directs whole-body regeneration as well as lineage restriction of replacement cells in various regenerating organs. Several cell signaling pathways known to function in regeneration in other animals are absent from the ctenophore’s genome. Further research will either reveal ancient principles of the regenerative process common to all animals or reveal novel solutions to the stability of cell fates and whole-body regeneration.

## 1. Introduction: Ctenophores Are a Key Model for Understanding Animal Regeneration

Regeneration is common across the animal kingdom—so common that it was likely present in the last common ancestor of all multicellular animals [1]. However, despite this apparent common origin, its manifestations are incredibly diverse, with varying limitations and cellular and molecular mechanisms among species. Which of these reflect the ancestral condition remains unclear. Furthermore, numerous independent losses of the trait suggest that, despite its apparent adaptive value from our anthropocentric viewpoint, it does not convey such a significant fitness advantage as to be highly conserved. Alternatively, “regeneration” may not be a single, homologous trait that arose early in multicellular animals but an epiphenomenon of other processes such as normal development, growth, and homeostasis. In this case, the apparent similarities underlying regeneration in different taxa would arise from the conservation of those underlying processes but “regeneration” per se would have arisen independently multiple times. Knowing which of these is the case will be essential to any fundamental understanding of regeneration.

**Figure 1:**
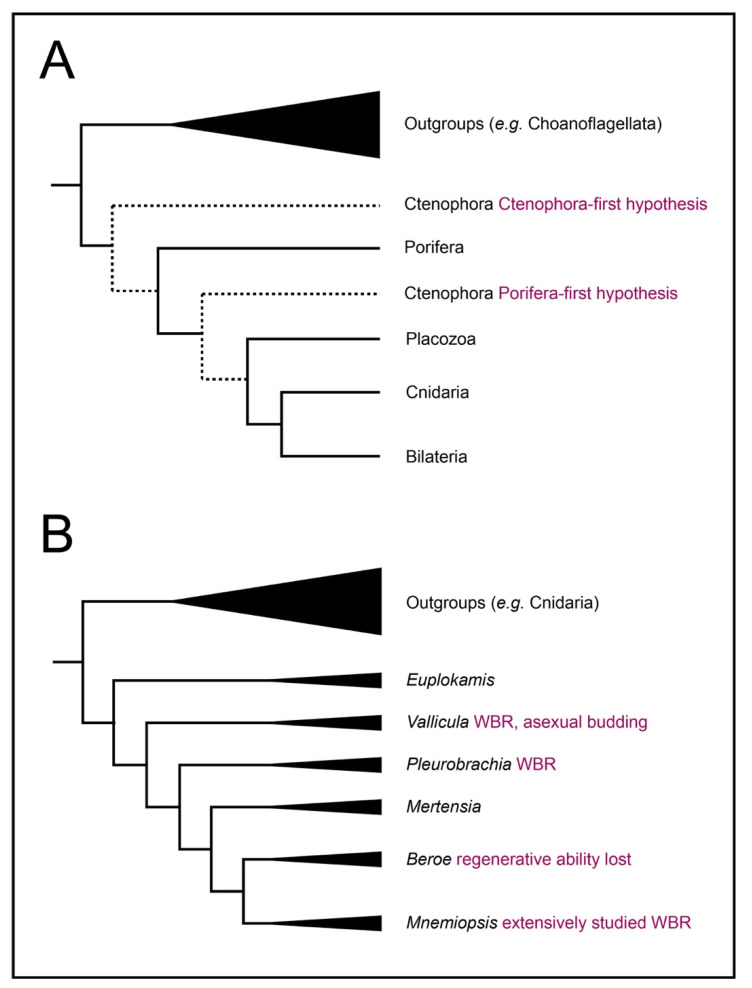
Evolutionary relationships of ctenophores. Topology drawn from [7]. (**A**) Relationships of metazoans. Ctenophores are the sister to all other metazoan taxa. (**B**) Evolutionary relationships among selected ctenophore genera; branch tip labels include genera referred to in this manuscript and its cited sources. Note that revision of traditional orders has been strongly suggested by most recent reconstruction, and position of Mertensia is not fully resolved.

Ctenophores are a classical model system for regeneration as well as embryonic development and are conveniently positioned to be informative about the evolution of regeneration (Figure 1). Most ctenophores exhibit extensive regeneration throughout their lives; many can replace with perfect fidelity not only a single missing organ but half or more of the structures in the body [2,3]. Only one lineage of ctenophores, the Beroids, has lost the ability to regenerate—indicating that it was present in the most recent common ancestor of all ctenophores (Figure 1B). Moreover, within the ctenophores, a range of regenerative capacities can be found. Benthic ctenophores, such as the platyctene *Vallicula*, can bud small pieces of peripheral tissue lacking any particular organ that nevertheless generate a complete animal [4]. 

Ctenophores exhibit diverse and complex phenotypes in body plan, cell type, ecological niche, and physiology, and this taxon is crucial for understanding animal origins [5,6]. The phylogenetic placement of ctenophores allows them to serve as an outgroup to all other animals commonly used for regeneration studies regardless of whether one considers the Ctenophora-first or Porifera-first phylogenetic hypothesis [7,8,9,10]. If there are deeply conserved core elements common to regenerative mechanisms, ctenophores are ideally positioned to identify them Figure 1A).

However, what is currently known about ctenophores problematizes our understanding from other animals in several ways. Established models for studying animal regeneration fall into two broad categories: whole-body regeneration by means of dedicated pluripotent stem cells and organ-level regeneration by dedifferentiation of terminally differentiated cells and redifferentiation into a different cell type to replace missing structures (reviewed in [1,11,12]). Ctenophores seem to defy the classical distinction between limited regeneration by local dedifferentiation and extensive, whole-body regeneration by dedicated pluripotent stem cells. Ctenophores also appear to lack several cell signaling pathways that have been proposed as potential universal regulators of regeneration in the active quest for commonalities in animal regeneration [13,14,15,16]. Thus, understanding how ctenophores regenerate will be essential to confirming whatever universal aspects of regeneration may exist.

## 2. Regeneration in Ctenophores

### 2.1. Ctenophores Have Diverse Cell Types and Structures, All of Which Can Regenerate

**Figure 2:**
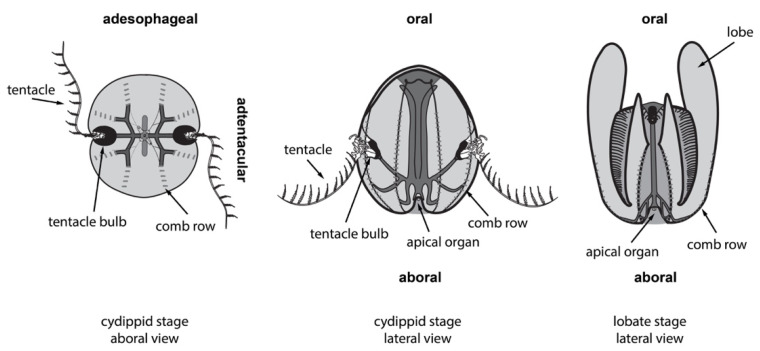
Body plans of the ctenophore *Mnemiopsis leidyi*. The major body axes (oral, aboral, adesophageal, and adtentacular) are labeled in bold for each view/stage. Their early postembryonic life is spent in the cydippid stage (left side). They spend most of their adult life in the lobate stage (right side). The overall morphology and body plan of both stages are similar, and the transition occurs gradually as they grow.

The best-studied ctenophore is the lobate species *Mnemiopsis leidyi*. (Papers published prior to the systematic revision of the genus [17] may also refer to it as *M. mccraydyi.*) This species has two morphologically distinct forms depending on its life stage (Figure 2), the so-called cydippid ‘larval’ stage and the ‘adult’ lobate stage, but both maintain the same set of axes, cell types, and organs (although there may be some yet uncharacterized differences, such as putative epidermal sensory structures that appear on some lobates). 

Ctenophores have an oral–aboral primary body axis and secondary axes of biradial symmetry, i.e., two axes of symmetry [18] called the tentacular axis and the esophageal or sagittal axis. Thus, adjacent quadrants contain different structures while opposing quadrants contain the same set of structures. In lobates such as *M. leidyi*, the cydippid body plan is a juvenile form in which the animal uses a pair of adhesive tentacles to catch prey; in the lobate stage, these tentacles are borne in shallow grooves internally to a pair of oral lobes. Both cydippid and lobate stages are equally capable of extensive regeneration to replace any of their organs and structures after injury [2]. While all available data point to regeneration at juvenile (cydippid) and adult (lobate) stages as being identical, it remains formally possible that there are differences in the underlying molecular mechanisms.

Ctenophores possess numerous cell types including an outer epidermis, a complex digestive system comprising a pharynx connected to highly ramified endodermal canals that circulate nutrients and two anal pores that eliminate waste [19,20,21,22], multipolar neurons, several distinct kinds of muscle cells, and several kinds of incompletely characterized mesenchyme such as the stellate cells within the watery network of mesoglea that makes up the bulk of its body. They have several complex organs, including an aboral sense organ (“apical organ”) which includes a gravity-sensing statocyst comprising mechanosensory cilia and a group of living biomineralized cells that deflect the balancer cilia depending on orientation to gravity [23] as well as other putative specialized sensory cell types such as photocytes [24]. Each cluster of balancer cilia is directly connected to the locomotory system of longitudinal comb rows made up of ciliary comb plates (or ctene plates, from which ctenophores take their name) for one quadrant of the biradial body plan [25]. The tentacle bulbs from which the tentacles emerge are complex organs that evidently contain large numbers of proliferative cells, likely to enable rapid turnover of the sticky colloblast cells, nerves, and muscles of the tentacles. 

### 2.2. Classic Experiments Demonstrated Ctenophores’ Capacity for Extensive Regeneration

A very early observation that after an intensely stormy period only fragments of ctenophores could be collected for some time [26] provided the earliest hint that ctenophores might regenerate. However, ctenophores’ regenerative abilities were not suspected until much later because they were known primarily as models of mosaic embryonic development. The earliest apparent demonstration of ctenophores’ regenerative capacity was when a few *Bolinopis infundibulum* damaged by collecting nets were cultured for several days until they regrew their lost oral lobes [27]. Detailed observations of *M. leidyi* during regeneration of missing parts later established their key abilities and constraints: a series of experiments by B. R. Coonfield showed that lobate stage animals could regenerate excised structures, such as a few comb rows or the apical organ [28]; could replace half their complement of body parts when bisected along the primary body axis [3]; could regenerate from even smaller fragments on occasion when cut into thirds or quarters, although smaller pieces were not successful; and could continue to live normally with half the full complement of normal structures in incompletely regenerated animals. Furthermore, this same paper showed that wound healing is extremely rapid, and that the presence of an intact apical organ greatly improves the odds and speed of complete regeneration. A new apical organ is the first organ to appear in successful regeneration of pieces missing that organ [3]. Thus, the understanding that the apical organ is an organizing center for axial patterning during regeneration was established from these elegant early experiments simply by cutting lobate stage *M. leidyi* along different planes. These observations have been confirmed and extended to earlier stages in the *M. leidyi* life cycle by subsequent investigations.

Surgically removed pieces of *M. leidyi* can regenerate any cell type or organ, regardless of whether those same types or organs are present in the operated piece. For example, the apical organ can be removed, and the oral piece faithfully replaces the apical organ including balancing cilia, lithocytes and dome cilia not present in the oral piece [2,3]. While there is a minimum amount of the body required to successfully regenerate, the minimum complement of cell types and amount of tissue required to regenerate is not known exactly. Finally, these early experiments showed that wound healing is extremely rapid in ctenophores, and this has since been confirmed with modern techniques. Even quite large wounds, such as complete bisection of the animal, close within 5 h in large lobates [3] and 1–2 h in cydippids of [29]. After a puncture injury, a network of filopodia close the wound in minutes. Time-lapse movies show that cells in the mesoglea migrate to the wound site and participate with epithelial cells at the wound margin to mechanically close the wound [29].

### 2.3. Regeneration Experiments Demonstrate a Global Axial Patterning System in Adult Ctenophores

As mentioned above, ctenophores’ extensive capacity for regeneration as adults contrasts with highly mosaic development. A bisected embryo becomes two stable half animals, each maintaining half the normal complement of body structures (e.g., four comb rows, one tentacle bulb, and so on) [2,26,30]. This happens when the embryo is bisected any time between first cleavage and about 15 h post-fertilization (hpf) [2]. After this stage, *M. leidyi* attains its extraordinary capacity for whole-body regeneration. However, the molecular basis for this dramatic change in capacity to respond to surgical intervention remains unknown.

**Figure 3:**
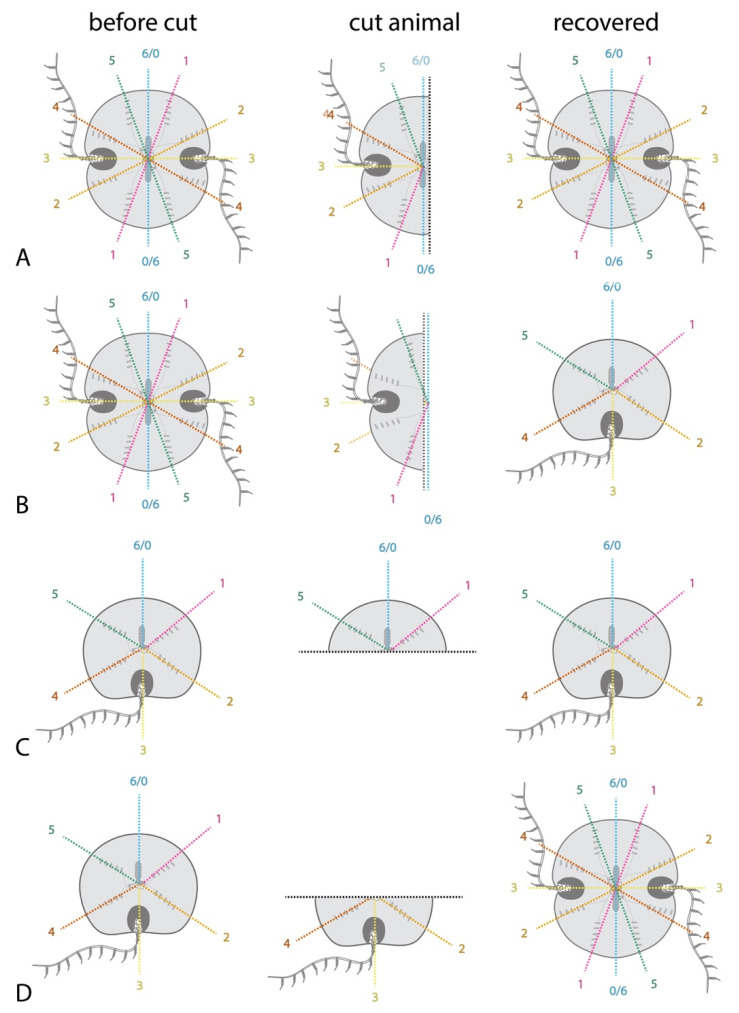
The apical organ serves as an organizing center to pattern the body axes during regeneration. The polar coordinate system of the ctenophore’s biradial body plan is indicated with differently colored lines for each plane of mirror symmetry; the identity of these planes is conventionally designated with a number as shown. (**A**) When a normal animal is bisected sparing the apical organ, it most often regenerates into a whole animal with two of each body part. (**B**) When the apical organ is not present, the bisected half may regenerate into a normal animal as in (**A**) but is more likely to become a stable half animal with one (instead of two) of each body part and no planes of mirror symmetry. The half-animal includes one tentacle bulb, one anal pore, half an apical organ, and half the normal complement of comb rows. (**C**) A “stable half-animal”, as generated in (**B**) or through embryonic deletion of half the body structures, can be bisected again. The fragment with the retained “half apical organ”, regenerates into a half animal. (**D**) In contrast, the fragment of the “half animal” lacking any apical organ can regenerate into an animal with all the body parts and axes of mirror symmetry present in a normal animal, including a whole apical organ. These results strongly suggest that the apical organ directs patterning when present. Removal of a half apical organ, which would otherwise direct patterning into a half-animal, allows the animal fragment lacking an apical organ to re-pattern normally (as a minority of animals cut as in (**B**) would do).

Regeneration is most likely to be successful when the aboral sense organ (“apical organ”) is intact or the apical organ itself is neatly removed but the longitudinal axis is undamaged [4,18]. This suggests that rather than establish a de novo signaling center to polarize regenerating cells, the ctenophore uses the apical organ as a central point to identify the original axes. This polar coordinate system of body patterning is reviewed in [31] and illustrated in Figure 3. Quartered animals reliably regenerate into stable half-animals, as do many fragments lacking the apical organ when animals are cut in half. Half-animals can live more or less normally. It appears that half animals are stable because cells belonging to a quadrant sense whether they are next to an opposite quadrant and regeneration of the missing quadrant identities is initiated only if this is not the case. Thus, a half animal’s cells cannot tell that it is incomplete because it contains all coordinate identities in their normal relative orientations. Whole animals can be generated from half animals by the addition of cells on the side opposite the cut site [2].

A stable half animal generated by bisecting embryos before the onset of regenerative ability will have a half apical organ, with two groups of balancer cilia to control only two body quadrants rather than the typical four in unmanipulated animals. If this animal is later cut, a fragment containing the intact half apical organ will regenerate into a half animal, while a fragment lacking any apical organ may regenerate into a whole animal [2] (Figure 3). This shows that the apical organ’s symmetry properties control the overall body plan during regeneration if it is present. This is one of the first demonstrations in any organism that defects arising during embryogenesis can be repaired by a later regenerative response. However, when the apical organ is missing peripheral structures will influence patterning during regeneration. If the apical organ is removed from normal adult animals, as expected, a normal apical organ with four groups of balancing cilia is produced. However, if the apical organ of a half animal is removed, a new ‘half’ apical organ is regenerated, possessing only two groups of balancing cilia. This indicates that the complement of existing tissues influences the patterning of the apical organ during its regeneration. 

**Figure 4:**
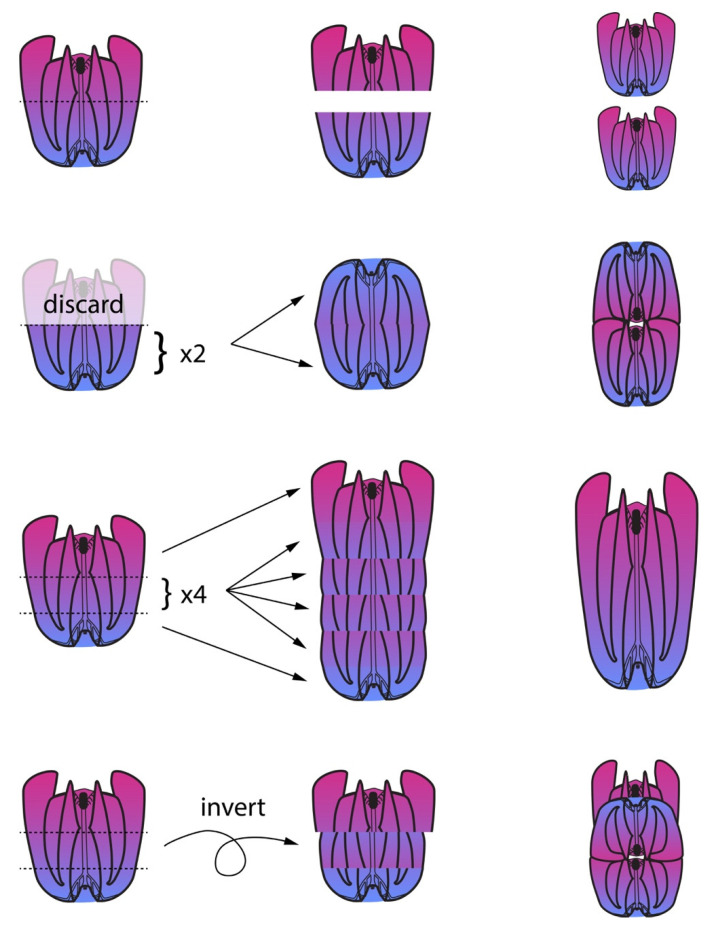
Cut-and-paste grafting experiments by Coonfield show that the oral–aboral axis is stably determined in adult ctenophores. **Top row**: animals bisected equatorially regenerate into two normally patterned animals. **Second row**: when two aboral halves are grafted together, both retain their original polarity. Each half grows new oral tissues such as mouth and lobes and the animals are fused at the oral region. **Third row**: when animals are cut into thirds and a series of middle pieces from different animals (four individuals in this case) are grafted in their native orientation with one apical and one oral piece, the animal regenerates to have a single common oral–aboral axis. **Fourth row**: when animals are cut into thirds and the middle piece is inverted on the oral–aboral axis, all three pieces maintain their original polarity (although the oral piece may not regenerate a new apical organ.

Transplantation experiments show that positional identity along the oral–aboral axis is fixed (Figure 4). First, when an animal is bisected equatorially into oral and aboral halves, each half can regenerate into a normally patterned animal [3]. (The same is true when cut into thirds parallel with this same axis.) When two animals are similarly bisected and their aboral halves are grafted together at the cut side, each half maintains its original axial pattern, resulting in a conjoined oral opening [18]. However, when animals are cut into thirds to produce an aboral piece, an oral piece, and a middle piece, the middle pieces of several animals can be stacked together with oral and aboral pieces at each end to produce a normally patterned animal with a single primary axis, so long as all the pieces are maintained in their original orientation [18]. However, if the middle piece is grafted between an oral and aboral piece at 180° from its original orientation (i.e. inverted on the oral-aboral axis), it produces a similar result to the grafting of two aboral ends together. Namely, the middle piece retains its original axial patterning and so it regenerates an apical organ to produce two animals fused near the oral opening (although the oral third apparently rarely recovered in these experiments); the alimentary canals of all three pieces can fuse [22]. 

Comb row transplantation experiments further show that ctenophores’ cells have a fixed oral–aboral polarity: if a portion of one of the comb rows is removed, rotated 180° along the oral-aboral axis, and reimplanted in its original place, over the course of several days the graft and host tissues will reorient so that the host’s intact comb rows meet up with the graft comb rows in their original orientation if possible or else avoid the graft to meet one another. The regenerated comb row may swerve across the body so that it bends rather than remain parallel to the primary body axis to preserve the oral–aboral directionality of the ctene plates’ original sequential identity.

### 2.4. Several Requirements for Ctenophore Regeneration Are Known: Developmental Stage, Nutrition, and Cell Division

The mere absence of a structure is clearly insufficient to initiate regeneration, or else the juveniles resulting from embryos with experimentally ablated cell lineages would replace these parts upon attaining competence to regenerate. However, at least three requirements for regeneration are known.

First, as mentioned above, *M. leidyi* exhibits a discrete switch point where the highly mosaic embryo becomes competent to regulate and regenerate extensively. Second, at least one environmental factor has also been shown to affect *M. leidyi*’s capacity to regenerate: food availability [32]. In particular, the abundance of food in the environment prior to the injury appears to confer competence to regenerate. In low-food conditions, bisected *M. leidyi* are much more likely to persist as half animals following wound healing; similarly, animals or animal fragments missing the aboral sense organ are much less likely to replace it in low food conditions. Interestingly, in a small percentage of cases, these animals can regenerate later, which further supports the observation that injury and repair can be temporally separated in these animals. It also suggests a degree of plasticity but does not resolve whether regeneration is a byproduct of another process, such as robust normal growth, or an adaptive trait. Third, cell division is indispensable for ctenophore regeneration. When cell proliferation is inhibited with hydroxyurea, experimentally cut animals can complete normal wound healing but cannot regenerate missing structures [29]. The same experiment thus demonstrates that wound healing is temporally separable from regeneration as regeneration can be initiated days after the injury if cell proliferation is permitted to resume by washing out the hydroxyurea. This suggests that injury response (including immune and stress responses) do not seem to be the direct cue for regeneration. 

### 2.5. Ctenophore Regeneration Does Not Appear to Use Dedicated Pluripotent Stem Cells

The mosaic embryonic development of *M. leidyi* was leveraged to ask whether pluripotent stem cells analogous to i-cells or neoblasts are involved in the replacement of missing structures in regeneration. Embryonically ablated comb rows and tentacle bulbs can be replaced in what has been called “post-regeneration” but the progenitor cells that participate are clearly lineage restricted [33]. The source of the replacement cells turns out to be exclusively from lineages that normally contribute to comb plates [34] or tentacle bulbs [35], respectively. Lineage restriction of these juvenile replacement cells suggests that there is likely not a pool of pluripotent stem cells that contribute large numbers of cells to regenerated structures.

There is some evidence that individual cells migrate to the site of injury, including direct time-lapse video evidence that cells in the mesoglea migrate to the wound site and participate with epithelial cells at the wound margin to mechanically close the wound. It is not yet clear if any of these migratory cells contribute to replacement structures or if their involvement is limited strictly to the healing process. Cells local to the injury site appear to contribute most of the cells to regenerated structures [29].

Regions of high stem cell density were hypothesized to serve as reservoirs for multipotent progenitors activated during regeneration. The tentacle bulbs are the organs with the highest number of stem cells as they continuously generate new tentacle tissue throughout the life of the animal. The tentacle bulbs thus, naturally, were suspected to contain neoblast-like cells. Combined with the observation that the ctenophore lineage with reduced ability to regenerate, the Beroids, also lacks tentacles, researchers were tempted to speculate that tentacle bulbs were necessary to regeneration. (While a single published observation has been cited in several places to claim that Beroids may be regenerative, the original source contains a single sentence about this taxon that draws on no specific, reported observations: “Judging from observations made in the Trondhjemfjord 1911 I cannot doubt that *Beroe cucumis* is also capable of regeneration to a large extent” [27].) However, the tentacle bulbs are not required for the regeneration of any structure, including the tentacle bulbs themselves; regeneration of the tentacle bulbs themselves and distant tissues alike proceeds normally after tentacle bulbs are excised [29]. It appears that tentacle bulbs’ stem cells are necessary only during normal growth and homeostasis to produce new colloblasts, the sticky cells on the tentacles, that are continually renewed on a sort of “conveyor belt” [36] and not for regeneration of distant organs. Finally, while apparent stem cells are present in adult *M. leidyi*, there is no evidence to suggest neoblast-like pluripotent cells are present, despite extensive lineage tracing [37]. 

The absence of dedicated pluripotent cells leaves a few possibilities. Cells might dedifferentiate but only to a limited extent and thus retain a preference for replacing cells from only certain lineages, as in, e.g., axolotl regeneration [38]. Alternatively, ctenophores may set aside lineage-restricted but multipotent stem cells during normal development that can be stimulated to generate new cell types upon the initiation of a regenerative event. Finally, it could be that some or all ctenophore cells have the capacity to dedifferentiate and reform the appropriate cell types in the appropriate locations when needed. 

### 2.6. M. leidyi Genomic Information Eliminates Several Candidate Cell Signaling Pathways

If a capacity for extensive regeneration is ancestral and the diverse modes of regeneration seen in animals are homologous, obscuring variation that has accumulated along different lineages obscures these core pathways. So far, there is not a single apparently universal aspect of animal regeneration. However, there are common threads shared by many systems investigated so far, such as Wnt signaling [39,40,41,42,43,44,45]; apoptosis [46,47,48]; innervation [49,50,51,52,53,54,55,56]; and cell proliferation [57,58,59], from either dedicated stem cell populations or partially dedifferentiated precursor cells. Numerous other potential cell signals affect regeneration including bioelectric signals [60,61,62] and signaling pathways such as MAPK [16,63,64,65,66,67] and target-of-rapamycin (TOR) [68,69,70,71,72]. 

Based on genomic data alone [10,73], it appears that we can eliminate at least some of these putatively shared mechanisms—particularly some cell signaling pathways—because the members of a functional pathway cannot be found in the genome (see Box 1). Due to its importance as a classically studied model organism for developmental biology and regeneration and as an ecologically and economically significant invasive species [74], excellent genomic resources are available for *M. leidyi* in particular (Box 1). These molecular observations are also supported by resources generated from a few other species [73,75]. Ctenophores possess members of some cell signaling pathways and homologs of transcription factors known to operate in regeneration in other systems, but also lack some genes prominently associated with regeneration. For example, the FGF and Hedgehog pathways appear to be missing from ctenophore genomes [10]. Thus, despite their deployment in other animals during regeneration [76,77,78,79,80], these pathways cannot be part of a universal regeneration program since they are missing from the genome of a highly regenerative ctenophore. However, no specific cell signaling pathway has been empirically shown to be necessary for regeneration in ctenophores so the degree of overlap with regeneration in other systems is yet unknown.

Box 1 **Box 1.** Published *Mnemiopsis leidyi* genomic resources [10,77,81,82,83,84] and the cell–cell signaling pathways that are likely present or absent (based on whether putative orthologs of listed pathway members were identified in the reference genome). The absence of some cell signaling pathways that are important for regeneration in other metazoans in the highly regenerative *M. leidyi* narrows the search area for putative universal regulators of regeneration.

               
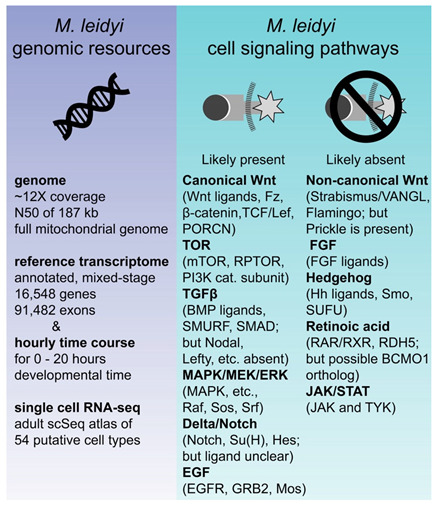

**Figure 5:**
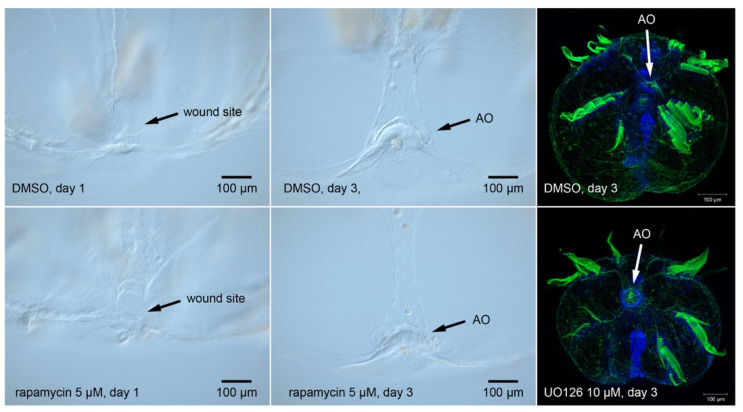
The TOR pathway inhibitor rapamycin and the MEK pathway inhibitor UO126 do not prevent regeneration in the ctenophore *M. leidyi*. Treatment with these inhibitors does not alter the time to replacement after amputation of the aboral sense organ (apical organ). Similar negative results appear to be widely known through word of mouth but seem to have remained unpublished elsewhere, so we demonstrate them here.

MAPK signaling in particular has been proposed as a potentially universal aspect of regeneration, as it is found in wound healing and regeneration phases of diverse animals [16,64]. While ctenophores possess members of the ERK/MEK signaling pathway (Box 1), we have been unable to produce a phenotype in *M. leidyi* when applying the inhibitor UO126 to regenerating *M. leidyi* cydippids (Figure 5). We obtained similarly negative results with the rapamycin, an inhibitor of the target-of-rapamycin (TOR) pathway, despite the presence of several key pathway members in *the M. leidyi* genome (Box 1) and its deployment in other animals during regeneration. We obtained similar negative results with small molecules that manipulate the Wnt pathway: LiCl, ICG-001, C59 (not shown).

As mentioned above, several other cell signaling pathways that are known to function in regeneration in other animals are missing from the *M. leidyi* genome. These include FGF, Hedgehog, and retinoic acid signaling. Some cell signaling must be implicated in ctenophore regeneration, so we hope future work can identify what pathways are used, or at least provide definitive evidence that the pathways we examined here are not used.

## 3. Future Directions

Ctenophores are an informative taxon for understanding the evolution of regeneration. In addition to whole-body regeneration, ctenophores combine several unusual features that make them potentially quite useful as a research organism. They are optically clear at all life stages, have a highly stereotyped mosaic development that permits precise cell lineage tracking and ablation, develop rapidly, and are direct developers so features manipulated embryonically (including microinjected materials) are retained. 

Most phyla have both examples of extensively and poorly regenerating species and ctenophores are no exception. While extensive regeneration appears obviously beneficial from our anthropocentric perspective, the many losses of this trait suggest it is not universally adaptive. Ctenophores also permit us to examine the loss of regeneration. The loss of regenerative capacity in the Beroid ctenophores will be of particular interest given their apparently conserved embryogenesis and sympatric distribution with highly regenerative species. Parallel investigations with *Beroe* will help us determine whether loss of regenerative capacity is a byproduct of another aspect of their biology. 

Several aspects of ctenophores’ regenerative program remain poorly understood. Major outstanding questions include: the degree of dedifferentiation vs. lineage restriction in replacement cells, whether any pluripotent stem cells contribute to regeneration, what cell–cell signaling and gene regulatory networks control regeneration, the role of the wound epidermis, and whether aspects of their regenerative program are shared with other models such as involvement of nerve cells. Basic descriptive work, including unbiased screens for differential gene regulation during regeneration (e.g., RNA-seq) will be an important step to identify candidates for functional studies, whether or not these turn out to be conserved with other models of regeneration. Such work can also form the basis for comparative studies with non-regenerative ctenophores to identify changes in gene regulation associated with failure to regenerate.

Histological data across species show that the apical organ is a site of dense innervation [85]. The reappearance of ciliated furrows which connect the locomotory system to the sense organ is the first morphological sign of regeneration in bisected animals. Similarly, the prospective aboral sense organ primordium is the first morphological structure to reappear during regeneration of the aboral region. These observations, coupled with the well-known involvement of the aboral sense organ in patterning regenerating half and quarter animals in *M. leidyi*, suggests that involvement of the nervous system might be crucial to ctenophore regeneration. Experimental manipulation of neural patterning, such as by embryonic ablation, may be able to confirm or rule out neural involvement.

Furthermore, the ability to regenerate in *M. leidyi* begins at nearly the same time that coordinated beating of the locomotory comb rows begins [2], suggesting that the nervous system must reach a key developmental stage for this ability. However, whether the nervous systems of ctenophores and other metazoans are actually homologous rather than convergent remains unresolved [10,73]. If the directing cell type has a similar function but is in fact a product of convergent evolution, this would raise new questions but might also confirm that sensory feedback is an essential part of regeneration in animals. 

Re-establishment of axial patterning is another extremely common element of regeneration. However, the molecular mechanisms of axial patterning in ctenophores remain less well understood than those of many other regeneration models. Learning more about the axial patterning mechanisms of embryonic ctenophores may also identify candidate genes to ask whether this step in regeneration is conserved. 

Given that Wnt signaling is frequently involved in regeneration across animals, it is surprising that inhibitors of the Wnt pathway have failed to produce regeneration or development phenotypes in ctenophores. Thoroughly eliminating this signaling pathway as a possible regulator of regeneration in ctenophores or finding a new approach to demonstrate its involvement seems essential to understanding whether regeneration is a conserved process with shared molecular underpinnings or an epiphenomenon of other biological processes. Furthermore, even if Wnt signaling is shown to be involved in ctenophore regeneration, its presence alone will not be sufficient to differentiate between homology and convergence. There is a ship-of-Theseus problem: convergent phenotypes are often reached by deployment of conserved tool sets, and there is no clear-cut answer to how many of the individual ligands, receptors, modulators, and transcription factors should be orthologous to feel certain of the true homology of two processes. 

Many recent regenerative studies have largely focused on integrating cell type diversity with gene expression in various taxa. Genes associated with development and germline maintenance are often identified as candidates in regeneration [86,87,88] notably the RNA interfering transposon silencer *piwi*, the DEAD-box RNA helicase *vasa* and the translational inhibitor *nanos*. In the ctenophore *Pleurobrachia pileus*, homologs of *vasa* and *nanos* are expressed in the adult gametogenic regions as well as highly proliferative cell clusters in the tentacle base, tentacles, and apical organ [89]. In *M. leidyi*, these genes are only expressed in the embryonic stage in areas of high mitotic activity with no expression in future gametogenic region [90]. Expression of these genes has not been assessed in adult *M. leidyi*, or during active regeneration of any ctenophore, so their function in adult regeneration remains speculative.

In addition to these pluripotency markers, regeneration often employs controlled re-deployment of portions of the developmental gene regulatory programs that specify and pattern the regenerating structures during their embryonic development. Further work to examine the expression patterns of tissue- and organ-specific lineage markers in regenerating ctenophores will help to clarify how much of the embryonic program is activated in regenerating ctenophores, although the extent to which regeneration is primarily a redeployment of developmental mechanisms appears to vary widely by taxa and regenerating structure. Finally, if ctenophores accomplish whole-body regeneration by means of lineage-restricted dedifferentiation, as appears to be the case, then they operate a unique regenerative program and may reveal fundamental cell regulatory mechanisms that would be of broad interest. The answers to these questions in ctenophores will influence our understanding of regeneration as a phenomenon.
